# Relation between thickness, crystallite size and magnetoresistance of nanostructured La_1−_*_x_*Sr*_x_*Mn*_y_*O_3±δ_ films for magnetic field sensors

**DOI:** 10.3762/bjnano.10.24

**Published:** 2019-01-23

**Authors:** Rasuole Lukose, Valentina Plausinaitiene, Milita Vagner, Nerija Zurauskiene, Skirmantas Kersulis, Virgaudas Kubilius, Karolis Motiejuitis, Birute Knasiene, Voitech Stankevic, Zita Saltyte, Martynas Skapas, Algirdas Selskis, Evaldas Naujalis

**Affiliations:** 1 Department of Material Science and Electrical Engineering, Center for Physical Sciences and Technology, Sauletekio av. 3, LT-10257 Vilnius, Lithuania; 2 Institute of Chemistry, Faculty of Chemistry and Geosciences, Vilnius University, Naugarduko 24, LT- 03225 Vilnius, Lithuania; 3 Department of Electrical Engineering, Faculty of Electronics, Vilnius Gediminas Technical University, Naugarduko 21, LT- 03227 Vilnius, Lithuania; 4 Department of Metrology, Center for Physical Sciences and Technology, Sauletekio av. 3, LT-10257 Vilnius, Lithuania; 5 Department of Characterization of Materials Structure, Center for Physical Sciences and Technology, Sauletekio av. 3, LT-10257 Vilnius, Lithuania

**Keywords:** colossal magnetoresistance, crystallites, magnetic field sensors, MOCVD growth, nanostructured films

## Abstract

In the present study the advantageous pulsed-injection metal organic chemical vapour deposition (PI-MOCVD) technique was used for the growth of nanostructured La_1−_*_x_*Sr*_x_*Mn*_y_*O_3±δ_ (LSMO) films on ceramic Al_2_O_3_ substrates. The compositional, structural and magnetoresistive properties of the nanostructured manganite were changed by variation of the processing conditions: precursor solution concentration, supply frequency and number of supply sources during the PI-MOCVD growth process. The results showed that the thick (≈400 nm) nanostructured LSMO films, grown using an additional supply source of precursor solution in an exponentially decreasing manner, exhibit the highest magnetoresistance and the lowest magnetoresistance anisotropy. The possibility to use these films for the development of magnetic field sensors operating at room temperature is discussed.

## Introduction

Perovskite manganite materials are an interesting topic of research since they can be applied as sensors for measuring the magnetic field due to the colossal magnetoresistance (CMR) phenomenon [1]. The complex physics of manganite materials provides an opportunity to tune their electric and magnetic properties over a wide range by variation of chemical composition [2–5], film thickness [6–7] and nanostructure [8–9], as well as induced lattice strain [10–12]. The manganite films consisting of columnar nanograins have already been successfully applied for the sensing of high pulsed magnetic fields (B-scalar sensor) [13–14]. Despite this development, the scalar (independent of field orientation) CMR effect under a low magnetic field is still a challenging goal towards practical applications due to low sensitivity and large magnetic anisotropy [15–16]. For this reason, the investigation and control of the magnetoresistive properties of manganite materials on the nanometer scale is of great importance. It was shown that the change of nanostructure by variation of deposition temperature influences the magnetic properties of the films [17]. The increase of the deposition rate also results in changes in the crystallite dimensions, leading to a higher number of nucleation sites [18]. In our research, the pulsed-injection metal organic chemical vapour deposition (PI-MOCVD) [19–20] was used to enable easy and reproducible control of the growth rate and nucleation site density by introducing the additional supply source of the precursor solution to the reaction chamber.

The novelty of our investigations concerns the growth of La_1−_*_x_*Sr*_x_*Mn*_y_*O_3±δ_ (LSMO) films on ceramic Al_2_O_3_ substrates in two different technological ways, resulting in different microstructure of the obtained nanostructured films. Such films have an advantage in comparison with the epitaxial films grown on monocrystalline substrates since they exhibit high magnetoresistance (MR) values over a broader temperature range [1].

In this study, we present the possibility to tune and to select the necessary properties of nanostructured LSMO films by changing the film thickness and microstructure in order to obtain higher sensitivity and lower anisotropy, important for magnetic field sensing.

## Results and Discussion

**Figure 1:**
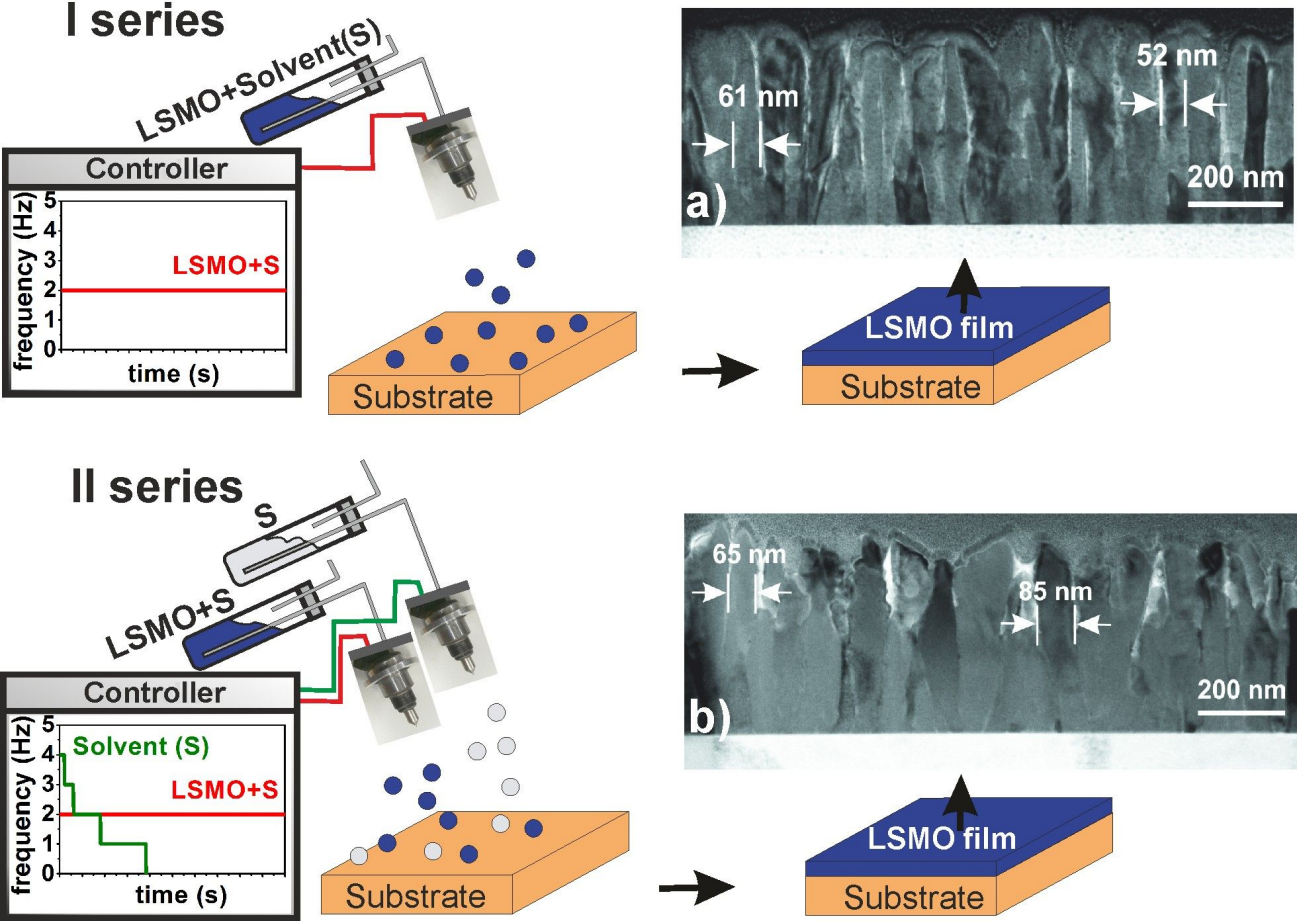
Schematic representation of the two deposition series of nanostructured LSMO films. I series – the mixture of precursors dissolved in the solvent is supplied to the reaction chamber. II series – 2 separate supply sources are used: i.) mixture of the precursor solution; ii.) solvent only, supplied in an exponentially decreasing manner leading to larger crystallites (TEM figures) and improved MR properties. (a, b) Cross-sectional TEM pictures of nanostructured LSMO films, for I and II deposition series.

Two series of films of variable thickness were deposited: I – one source with LSMO solution, II – 2 separate sources, LSMO solution and solvent source (Figure 1). The growth rate was controlled by application of additional solvent, resulting in the dilution of the precursor in the gas phase.

The transmission electron microscopy (TEM) analysis (Figure 1a,b) shows the column-like growth with larger dimensions and more dense, close packing of the crystallites for the II series.

### Film composition, structure and surface morphology

**Figure 2:**
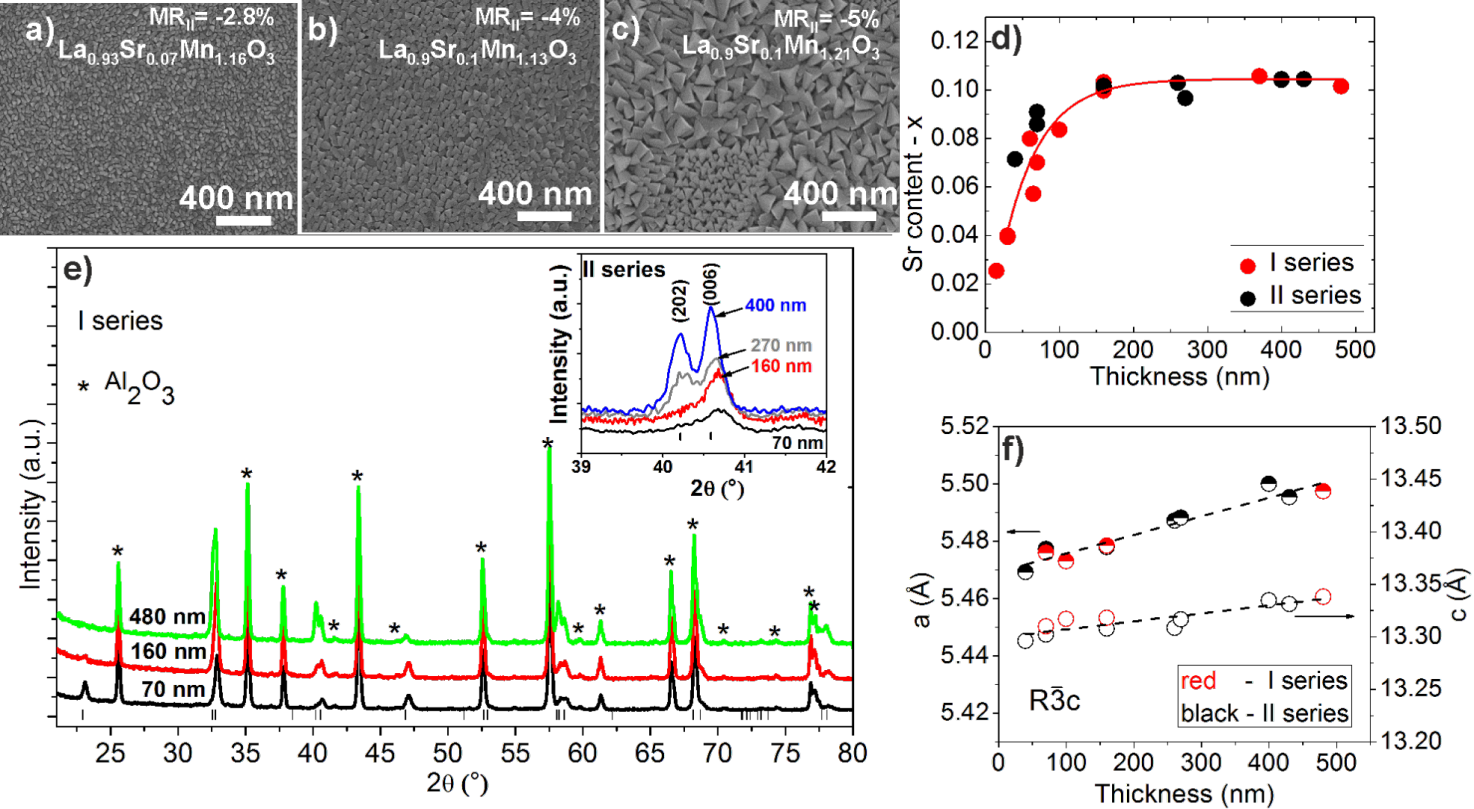
(a–c) SEM pictures of LSMO films (I series) deposited on Al_2_O_3_ substrates with different thickness: (a) 70 nm, (b) 160 nm and (c) 480 nm. (d) The average strontium (*x*) content (deduced from ICP-MS measurements) dependence on the thickness of the deposited LSMO films (I and II series, the red line is a guide for the eye). (e) GIXRD patterns for the LSMO films of different thickness for the I series. The inset presents peaks and their shift with thickness for the II series. The stars represent the characteristic peaks of the Al_2_O_3_ substrate, the vertical lines represent the characteristic peaks of LSMO in rhombohedral distortion. (f) The *a* and *c* lattice parameters calculated from XRD patterns for LSMO films of both series.

Figure 2a–c presents scanning electron microscopy (SEM) images of the films with different thickness showing the increase in the crystallite dimensions with the film thickness. Moreover, the mass spectroscopy measurements revealed the change of elemental composition: the average strontium amount decreased with the decrease of film thickness independent of the deposition series (Figure 2d). The amount of La (1−*x*(Sr)) was slightly decreased from 0.975 to 0.9 with respect to the increase of the amount of Sr from 0.025 up to 0.1. The measured content of Mn in the films was in the range of 1.12–1.21. The grazing incidence X-ray diffraction (GIXRD) measurements presented in Figure 2e show no secondary phases, only the characteristic peaks associated with the Al_2_O_3_ substrate and polycrystalline LSMO films with a perovskite-like crystal structure with rhombohedral distortions (the space group 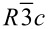) for both deposition series. The shift of the characteristic LSMO peaks to higher θ/2θ angles indicates the reduction of the *a* and *c* lattice parameters. The LeBail modelling of the XRD patterns showed the linear dependence of lattice parameters on the film thickness (Figure 2f). Additionally, the reduction of (*n*0*n*) peak intensities was observed for nanostructured LSMO films with a decrease of the film thickness for both deposition series (Figure 2e and inset). This effect is attributed to the reduction of the cell volume and appearance of strain in the films with the decrease of the film thickness, as was also observed by H. Baaziz and co-authors for La_0.9_Sr_0.1_MnO_3_ nanoparticles [21].

### Transport and magnetoresistive properties

**Figure 3:**
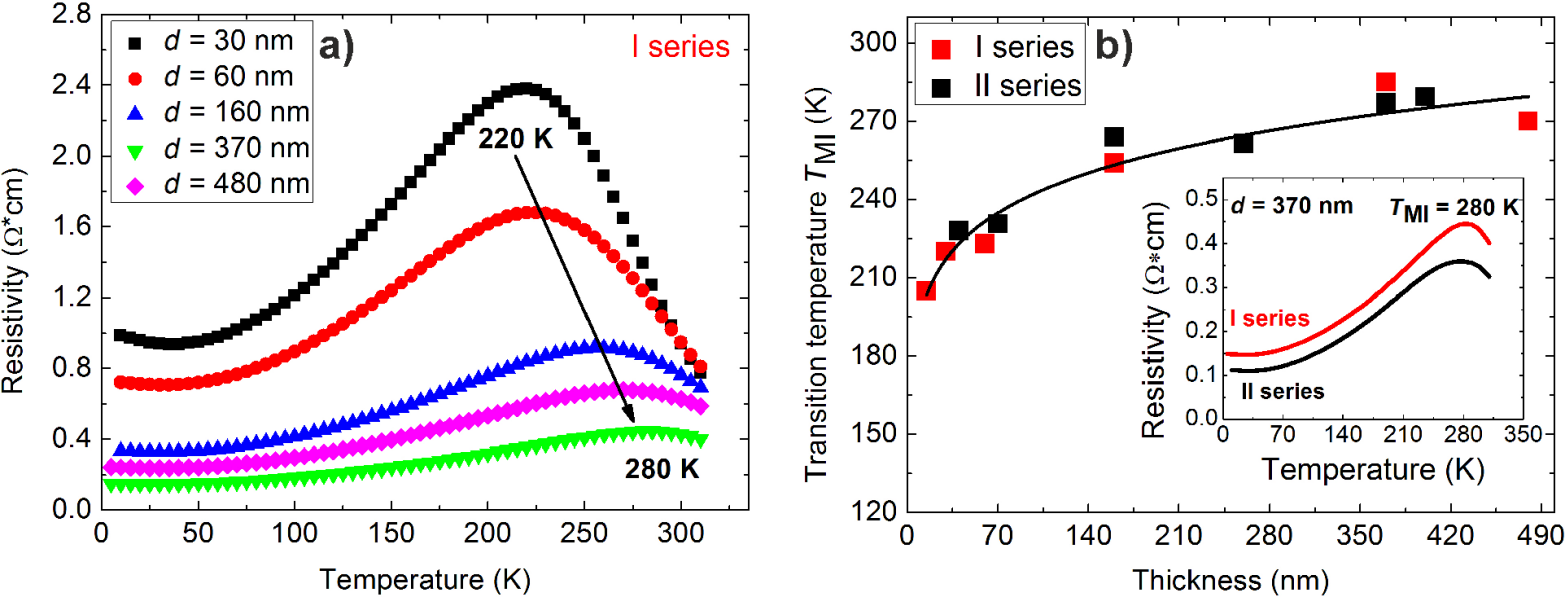
(a) Resistivity dependence on temperature for nanostructured LSMO films with thickness in the range of 30–480 nm grown on Al_2_O_3_ substrates; (b) *T*_MI_ dependence on film thickness for both deposition series, black line is a guide for the eye. Inset – resistivity dependence on temperature for 370 nm thick LSMO films of the I and II series.

In nanostructured manganite materials the difference in dimensions of crystallites and change of the relative amount of grain boundaries (GBs) and film composition significantly change the transport behaviour [17,21]. The decrease of electrical resistivity and the increase of the metal–insulator transition temperature (*T*_MI_) were observed with the increase of film thickness, crystallite dimensions and Sr content for both deposition series (Figure 3a and 3b). No significant difference in the *T*_MI_ was observed for the I and II series (Figure 3b insert).

**Figure 4:**
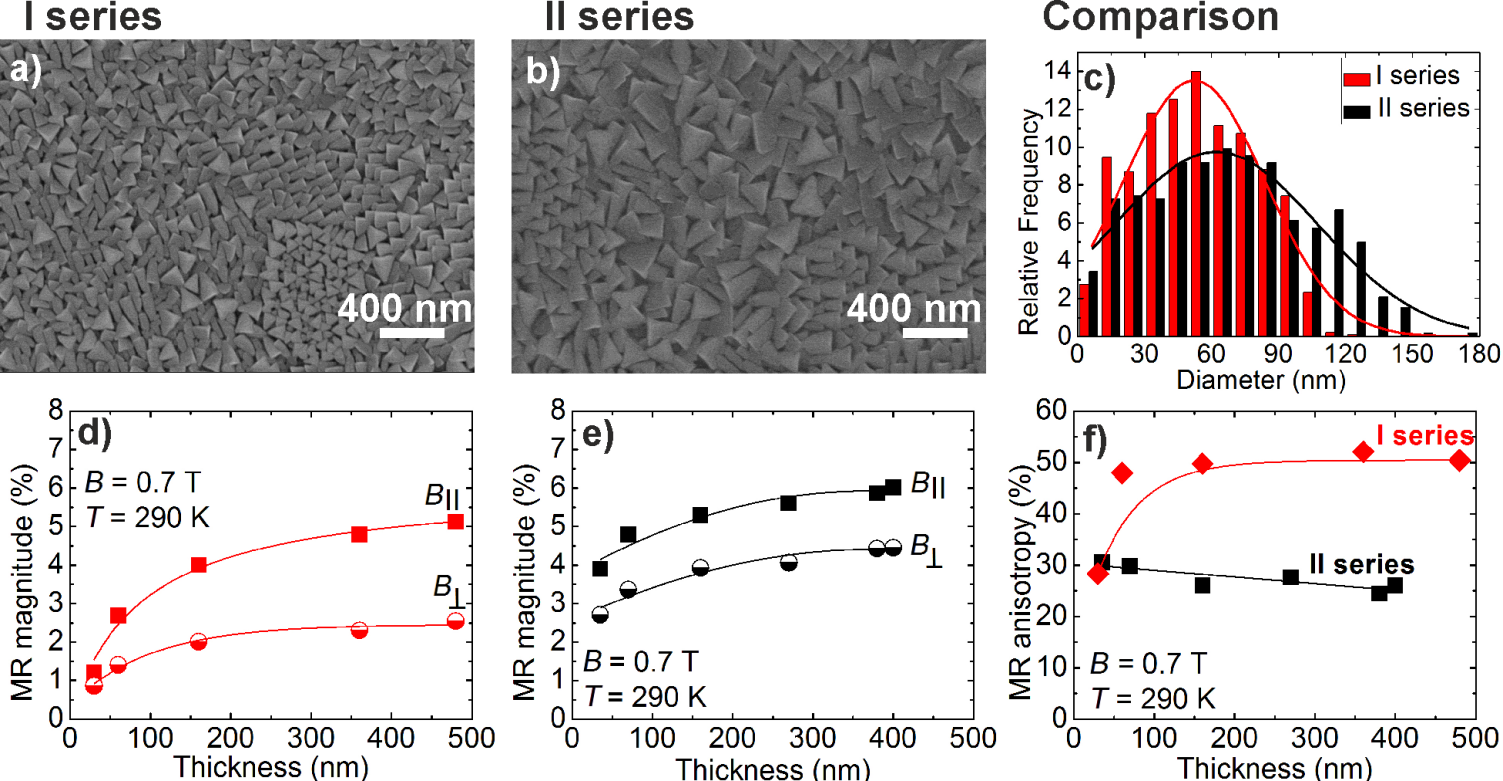
SEM picture of LSMO films grown from (a) one supply source - I series; (b) two supply sources - II series. (c) Comparison of relative frequency dependence on diameter of the crystallites for the I and II series 370 nm thick films. Magnetoresistance dependence on LSMO film thickness with applied external magnetic field of 0.7 T parallel (*B*_||_) and perpendicular (*B*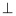) to the plane of the film grown from (d) one supply source and (e) two supply sources. (f) Magnetoresistance anisotropy dependence on film thickness for both deposition series. The lines are the guides for the eye.

However, higher resistivity values were observed for the I series films (Figure 3b insert) due to smaller crystallites (Figure 4a,b) and larger number of GBs. The average crystallite diameter of 56 nm and 69 nm was found for the I and II series (Figure 4c), respectively.

The technological processing and decrease of the growth rate (I series – 28 nm/min; II series – 18 nm/min) enabled an increase of the crystallite size at the same deposition temperature. In relation to the dimensions of the crystallites and transport properties, the increase of the MR (where MR = (ρ_B_ − ρ_0_)/ρ_0_ and ρ_B_ and ρ_0_ are the field and zero field resistivity, respectively) with film thickness was observed (Figure 4d,e). The largest MR magnitude (6%, when the field was directed parallel to the film plane *B*_||_ = 0.7 T) was obtained for the thickest films (≈400 nm) of the II series. The measured MR values of this II series films at room temperature are higher in comparison with the results obtained by other authors (<2%) [22–23]. For the field perpendicular to the film plane 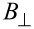, the MR was lower due to the demagnetization effect, which resulted in MR anisotropy 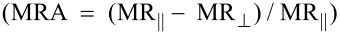 (Figure 4f). It is known that in thin manganite films the direction of the easy axis of magnetization is parallel to the film plane due to the fact that the demagnetization field is directly linked with the geometric shape of the sample [15–16]. In our case, this effect is partly compensated by the columnar structure of the film, where each individual crystallite has the easy axis of magnetization directed perpendicular to the film plane. Therefore, this compensation is more effective for thicker films of the II series, having larger monolithic crystallites with the most probably of higher individual magnetization in comparison with the films of the I series with smaller crystallites. As a result, the compensation of the demagnetization field leads to the lower MRA (25%) and slightly higher sensitivity ΔMR/Δ*B* (7.7%/T) measured at 0.7 T for II series films, whereas for the I series films, MRA is ≈50% and the sensitivity 6.5%/T. For higher fields, the MRA decreased (14% at 2 T, 2% at 10 T) implying the possibility to use these films for the development of B-scalar sensors operating under high magnetic fields.

## Conclusion

In this study, the nanostructured LSMO films were grown by PI-MOCVD in two different technological ways (with and without additional source of solvent) enabling the control of the microstructure and magnetoresistive properties of the films. It was demonstrated that the crystallite dimensions and magnetoresistance magnitude increase with the film thickness. Moreover, the usage of an additional solvent source decreases the growth rate of the films, leading to an increase of crystallite dimensions. As a result, an increase in the magnetoresistance and reduction of magnetoresistance anisotropy is achieved, which is technologically important for the production of magnetic field sensors.

## Experimental

The nanostructured LSMO films were grown on ceramic Al_2_O_3_ substrates by the PI-MOCVD technique by supplying a mixture of precursor solution and solvent in micro-doses of 3 mg. La(thd)_3_, Sr(thd)_2_, and Mn(thd)_3_ (where thd is 2,2,6,6-tetramethyl-3,5-heptandionate) were used as precursors and dissolved in the dimethoxyethane solvent. Two deposition series were performed using one (precursor solution – I series) or two (precursor solution and solvent – II series) precursor supply sources with a constant 2 Hz supply frequency. The software controlling the operation of the second supply source (solvent) is based on the following 5-step program: the supply frequency is kept constant during each step and is changed after different time intervals (steps) – 30, 90, 105, 280 and 350 s, in order to follow an exponentially decreasing law. During the first cycle, two additional micro-doses of solvent with respect to the precursor solution were supplied, whereas during the last cycle, only the precursor solution was injected. The supply of the solvent source during the intermediate cycles was varied as shown in Figure 1. The LSMO films were deposited at 750 °C and 10 Torr with a partial 3.5 Torr oxygen pressure and post-annealed for 10 minutes in oxygen atmosphere. The thickness of the films was changed in the range of 30–480 nm and determined using a Taylor Hobson Talystep profilometer. The crystal structure of the films was analysed by GIXRD measurements using a Bruker D8 Advance diffractometer, where the incident X-ray beam was fixed at 0.5°. The refinement of the peak shape in the XRD diffraction patterns was performed by using the computer program TOPAS 4.2. The XRD peak shape corrections were proceeded with LaB_6_ powder standard (SRM660a) certificated by the National Institute of Standards and Technology. The morphology of the films was investigated by SEM (Hitachi SU70). The structural analysis was performed in cross section geometry by TEM (FEI Tecnai G2 F20 X-TWIN). The elemental composition analysis was performed by inductively coupled plasma high-resolution mass spectrometry (ICP-MS) - Thermo Scientific Element2, where the films were totally dissolved in 2% nitric acid. For the electric transport and magnetoresistance measurements, the Ag contacts with a Cr sublayer were thermally deposited and postannealed at 450 °C for 1 h in Ar atmosphere. The magnetoresistance (MR) measurements were performed under a permanent magnetic field up to 0.7 T using an electromagnet and a pulsed field up to 10 T using a generator based on capacitor bank discharge through a special multi-shot magnetic field coil.
